# Exploring structural, mechanical, and thermoelectric properties of half-Heusler compounds RhBiX (X = Ti, Zr, Hf): A first-principles investigation

**DOI:** 10.1039/d3ra01262j

**Published:** 2023-04-12

**Authors:** Junhong Wei, Yongliang Guo, Guangtao Wang

**Affiliations:** a School of Science, Henan Institute of Technology Xinxiang 453003 China ylguo@hait.edu.cn weijh@hait.edu.cn; b School of Physics, Henan Normal University Xinxiang 453007 China wangtao@htu.cn

## Abstract

In this study, the full potential linearization enhanced plane wave method in density functional theory is used. Additionally, the structure, mechanical, and thermoelectric properties of half-Heusler compounds RhBiX (X = Ti, Zr, Hf) are investigated for the first time. The indirect semiconductors RhBiTi and RhBiZr have 0.89 and 1.06 eV bandgap energies, respectively. In contrast, RhBiHf is a direct bandgap semiconductor with a bandgap energy of 0.33 eV. The thermoelectric parameters such as Seebeck coefficient, power factor, electronic conductivity, lattice thermal conductivity, electronic thermal conductivity, and figure of merit *ZT*, are studied with the semi-classical Boltzmann transport theory. When *T* = 300 K, RhBiTi, RhBiZr, and RhBiHf show small lattice thermal conductivities, *i.e.*, 10.60, 10.15, and 7.71 W mK^−1^, respectively, which are consistent with related other studies. The maximum *ZT* values of RhBiTi, RhBiZr, and RhBiXHf are 0.91, 0.94, and 0.79 at 900 K, respectively. Furthermore, we observed that RhBiX (X = Ti, Zr, Hf) alloy is a thermoelectric material with great potential.

## Abstract 2

Calculated phonon band diagram (a), Seebeck coefficient (b), power factor (c), electrical conductivity (d), lattice thermal conductivity and electronic thermal conductivity (e) and ratio ZT_e_ (f) of RhBiX (X = Ti, Zr, Hf) at different temperatures.

## Graphical abstract

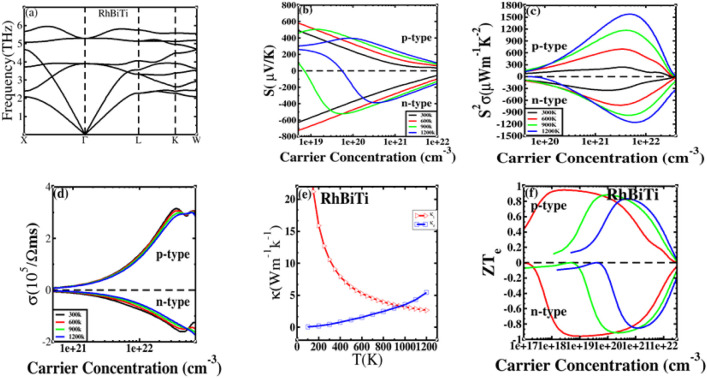

## 1. Introduction

Fuel shortage and environmental pollution are two major challenges in today's society. Dependency on fossil fuels is the primary reason for these issues. Therefore, exploring green and pollution-free energy materials and improving the energy conversion efficiency of materials is crucial.^1,2^ Thermoelectric materials, which can directly convert thermal energy into electrical energy, have received widespread attention because of their advantages in energy recovery.^3–6^ However, large heat waste takes place near ambient temperature, and the current application status of thermoelectric materials is that commercially used Bi_2_Te_3_-based thermoelectric materials have high cost and low conversion efficiency in the existing temperature range. This severely limits the large-scale application of thermoelectric materials.^7^ Therefore, novel thermoelectric materials with high performance and a wide range of elements must be explored.^8–12^ We observed that thermoelectric materials can be employed in daily life to meet the growing demand for energy. Highly efficient TE devices can generate electricity from heat waste.^13,14^

The conversion efficiency of thermoelectric materials can be measured in dimensionless figure of merit (*ZT*), which can be defined as follows: *ZT* = *S*^2^*σT*/*κ*, where *S* is Seebeck coefficient, σ is electrical conductivity, *T* is absolute temperature, and *κ* is total thermal conductivity. The total thermal conductance includes lattice (*κ*_ι_) and electronic thermal conductivities (*κ*_e_).^15,16^ Obtaining a high *ZT* value is complicated because in the formula that defines *ZT*, parameters, *σ*, and *κ* are interdependent and mutually restrictive. *σ* decreased with an increase in *S* value.^17–20^

Among thermoelectric materials, half-Heusler (HH) compounds have been recognized as promising thermoelectric energy materials suitable for medium and high temperatures.^21–24^ In addition, the elements contained in the alloy are nontoxic and environmental friendly. Therefore, ternary HH materials as thermoelectric materials have gained attention recently. FeNbSb exhibits an extremely high *ZT*, reaching 1.5 at 1200 K.^25,26^ For XIrSb (X = Ti, Zr, Hf), the reported values of *ZT* are 0.87, 0.95, and 0.90 for TiIrSb, TrIrSb, and HfIrSb at 800 K, respectively.^27^ XCoSb (X = Ti, Zr, Hf) compound gained attention due to their *ZT* value, which is equal to 1.0 at 1097 K in p-type doping.^28^ CuLiX (X = Se, Te) is considered an excellent thermoelectric material because of its high thermoelectric superiority value, where the value of CuLiTe (CuLiSe) is equal to 2.65 (1.7).^29^ For KBiX (X = Ba, Sr), based on theoretical calculation, *ZT* reached 2.68 (1.56) for KBiBa (KBiSr).^30^ J. Carrete used an *ab initio* method to screen a batch of structurally stable HH compounds and predicted that the materials had small lattice thermal conductivity.^31^ J. H. Liu applied a compressed-sensing approach to evaluate the lattice thermal conductivity with significantly high accuracy,^32^ where RhBiX (X = Ti, Zr, Hf) was within the range of their predicted HH compounds. The electronic structure and other physical properties of these materials have not been thoroughly studied. Therefore, we explored the electronic structure, mechanical properties, and thermoelectric properties of RhBiX (X = Ti, Zr, Hf) comprehensively and systematically by combining first-principle calculations and Boltzmann transport theory.

## 2. Methodology

We conducted simulations on HH compounds RhBiX (X = Ti, Zr, Hf) within density functional theory (DFT).^33^ The full-potential linearized augmented plane-wave (FPLAPW) in WIEN2K code was used to study the properties of materials.^34^ The Perdew–Burke–Ernzerhof generalized gradient (PBE) approximation^35^ and the project-augmented wave method were employed in our study. To obtain accurate band structures, we employed a modified Becke-Johnson (mBJ) potential.^36,37^ The plane-wave expansion cutoff energy in the wavefunction was set to 600 eV. For structural optimization, the standard for each ion convergence is set to less than 0.001 eV Å^−1^, and the convergence threshold set to 10^−7^ eV for total energy calculation in the electronic self-consistent. An energy threshold of −6.0 Ry was used to separate the valence and core states, and *R*_mt_ × *K*_max_ = 7, where *R*_mt_ and *K*_max_ represent the smallest muffin-tin radius and the magnitude of the largest reciprocal-lattice vectors, respectively. For the calculated self-consistency, we set the k-mesh to be 20 × 20 × 20 in the first Brillouin zone; for RhBiTi, the muffin-tin radii are set to 2.5 Bohr for Rh, 2.39 Bohr for Bi, and 2.5 Bohr for Ti. For RhBiZr, the muffin-tin radii are set at 2.5 Bohr for Rh, 2.46 Bohr for Bi, and 2.5 Bohr for Zr. For RhBiHf, the muffin-tin radii are set at 2.5 Bohr for Rh, 2.45 Bohr for Bi, and 2.5 Bohr for Hf. The phonon dispersion was calculated to investigate dynamical stability of the structures. This was conducted using a supercell approach^38^ as implemented in the PHONOPY code.^39^ Supercell size consisted of a set of 3 × 3 × 3, which is constructed from the optimized crystallographic primitive cell. The BZ integration was seted by a 2 × 2 × 2 k-point mesh. The forces induced by small displacements were calculated within Vienna *ab initio* simulation package (VASP).^40^ To determine the thermoelectric properties, we used the semi-classical Boltzmann transport theory with constant scattering time approximation (CSTA)^41^ as implemented in the BoltzTraP code^42,43^ and rigid band approximation (RBA). The thermoelectric properties of wide- and narrow-bandgap semiconductors^44–47^ have been studied with Boltzmann transport for a long time and recognized. With the BoltzTraP code, we could obtain the thermoelectric properties with 40 000 *k*-points of the dense *k*-mesh.

## 3. Structure stability and mechanical properties

**Fig. 1:**
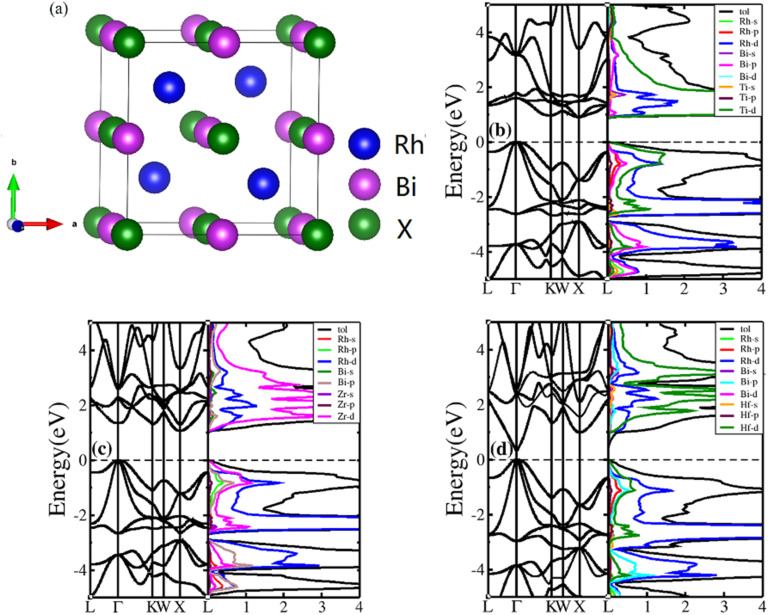
(a) The structure of the half-Heusler RhBiX (X = Ti, Zr, Hf). Band structure and projected density of states (PDOS) of RhBiX (X = Ti, Zr, Hf) (b) RhBiTi (c) RhBiZr (d) RhBiHf.

HH compounds exhibited a crystal structure of XYZ with space group F4̄3m (216)^48,49^ as shown in Fig. 1(a), where X, Y, and Z atoms were located in the Wyckoff positions of 4c (1/4,1/4,1/4), 4b (1/2,1/2,1/2), and 4a (0, 0, 0), respectively. For ternary HH compounds, three different atomic configurations theoretically exist, known as XYZ, YXZ, and ZXY. Results of first principles calculations demonstrate that the XYZ structure combination has the lowest energy. Additionally, no imaginary frequency exists in the phonon spectrum, implying that the XYZ-type compound is dynamically stable. First, we optimized the lattice constant. The optimized lattice constants were 6.265, 6.444 and 6.406 Å for RhBiTi, RhBiZr and RhBiHf respectively, which in good agreement with relevant theoretical research.^50^ With the optimized lattice constant, the band structures and projected density of states (PDOS) are calculated, as shown in Fig. 1(b)–(d). RhBiHf is a semiconductor with a direct band gap of 0.33 eV and the conduction-band minimum (CBM) and valence band maximum (VBM) located at the *Γ* point. RhBiTi and RhBiZr are semiconductors with indirect band gap of 0.89 and 1.06 eV with CBM and VBM located at the *X* and *Γ* points, respectively. We observed that the valence band is triply degenerate along *Γ*–*K* point. The greater the curvature of the bands, the smaller the effective mass of the corresponding carriers. The triple degenerate valence bands produce holes with three different effective mass values.

In the PDOS of RhBiX (X = Ti, Zr, Hf), we observed that near the Fermi level, it consists primarily of the d-orbitals of Rh, Hf (Ti, Zr) and the p-orbitals of Rh, Bi, Hf (Ti, Zr). The d-bands of Bi atoms were primarily distributed near the Fermi level and contributed by hybridized Rh-d and Bi-p bands. Therefore, from the band diagrams of RhBiX (X = Ti, Zr, Hf), we observed that the materials are p-type semiconductors.

**Fig. 2:**
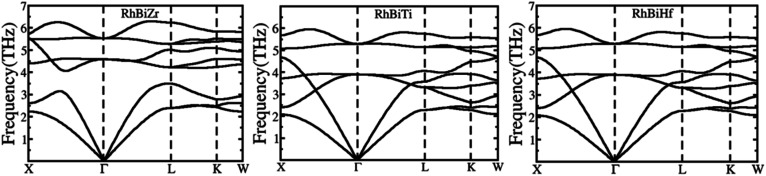
Phonon band diagram of RhBiX (X = Ti, Zr, Hf) compounds.

The phonon spectrums of RhBiX (X = Ti, Zr, Hf) are shown in Fig. 2. The high symmetry points are along X-Γ-L-K-W, and this showed that no negative phonon modes of frequencies existed, which implied that RhBiX (X = Ti, Zr, Hf) were dynamically stable. The three lower frequencies belong to acoustic modes, whereas the remaining six higher frequencies belong to the optical modes of RhBiTi, RhBiZr, and RhBiHf compounds. For RhBiHf and RhBiTi, the acoustic mode overlapped with the optical model, while for RhBiZr, the optical mode was separated from the acoustic mode. If the material had a small phonon band gap or maximum overlap, this reduced phonon scattering and obtained a lower lattice thermal conductivity.^51,52^ Thus, the overlapping of RhBiX (X = Ti, Zr, Hf) phonon modes indicates that the thermal conductivities of RhBiHf and RhBiTi are lower than that of RhBiZr.

The elastic parameters of (X = Ti, Zr, Hf) were calculated, which are listed in Table 1. In contrast to the elastic stability conditions,^53^ we can determine the mechanical stabilities of (X = Ti, Zr, Hf) compounds, which is stated as

1 *C*_11_ > 0,*C*_44_ > 0,*C*_11_–*C*_12_ > 0, and *C*_11_ + 2*C*_12_ > 0

From the elastic constants' relationship, RhBiX(X = Ti, Zr, Hf) satisfied the stability criterion. Based on the stability conditions of Born and Huang,^53^ the bulk modulus (*B*), shear modulus (*G*), Young's modulus (*Y*), the Poisson's ratio (*v*), longitudinal (*υ*_l_), transverse (*υ*_s_) velocity and average sound velocity (*v*_m_) are defined as^54,55^2
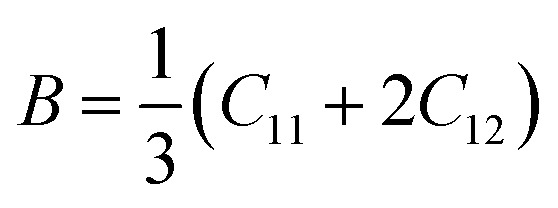
3
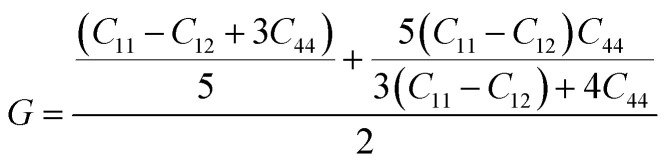
4
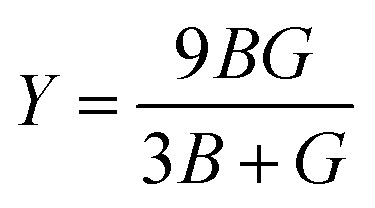
5
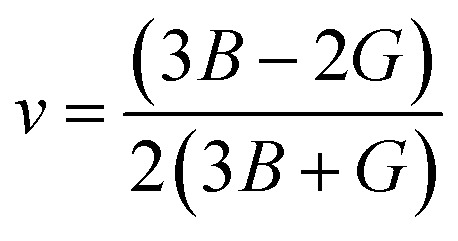
6
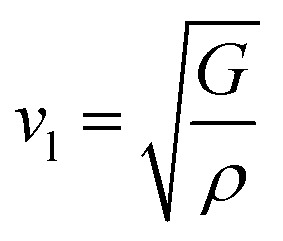
7
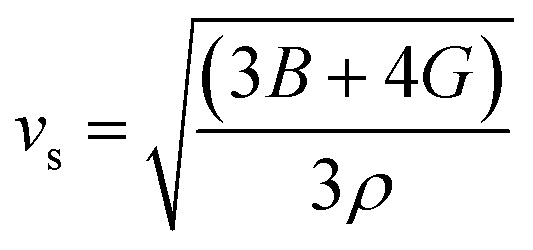
8
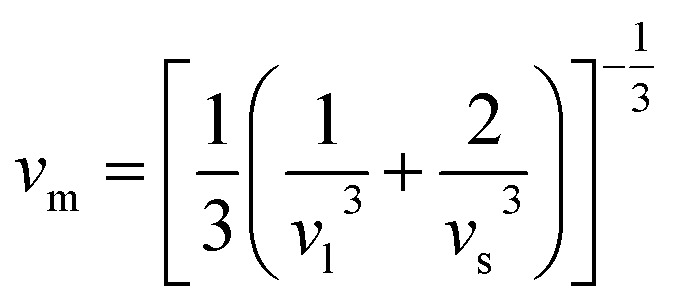


**Table 1:** Calculated the values of elastic (*C*_11_,*C*_12_,*C*_44_) (Gpa), bulk (*B*) (Gpa), Shear (*G*) (Gpa), Young's (*Y*) moduli (in GPa), Poisson's ratio (*ν*), *B*/*G* ratio, average sound velocity (*v*_m_) (ms^−1^), longitudinal velocity (*v*_l_) (ms^−1^), shear sound velocity (*vs.*) (ms^−1^), and Debye temperature (*Θ*_D_) (K) for RhBiX (X = Ti, Zr, Hf)

Parameter	RhBiTi	RhBiZr	RhBiHf
*C*_11_	181.18	194.16	197.07
*C*_12_	96.76	87.41	91.64
*C*_44_	37.20	50.18	46.77
*B*	124.90	122.99	126.79
*Y*	106.28	135.42	130.18
*G*	39.13	51.43	49.06
*B*/*G*	3.19	2.39	2.58
*ν*	0.36	0.32	0.33
*ν*_ι_	4268.69	4375.57	3938.88
*ν*_m_	2258.54	2537.74	2231.11
*ν*_s_	2006.60	2267.17	1990.08
*Θ*_D_	245.70	268.40	237.40

Debye temperature *Θ*_D_can be calculated by formula:^56^9
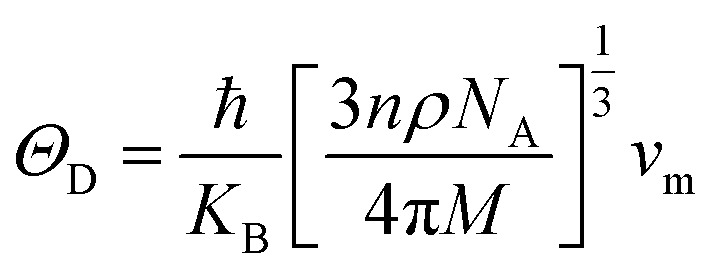
Where *ℏ*, *K*_B_, *n*, *N*_a_, and *M*are Planck constant, Boltzmann constant, number of atoms per unit cell, Avogadro's number, and atomic mass of the unit crystal cell, respectively. The ratio (*B*/*G*) can be used to determine the brittleness and ductility of the alloy.^57^ When *B*/*G* was <1.75, it indicated brittleness of the material, and *vice versa*, it indicated ductility. For RhBiX (X = Ti, Zr, Hf), the values of *B*/*G* were >1.75, thereby showing ductility.

## 4. The spin–orbit coupling (SOC) effect

**Fig. 3:**
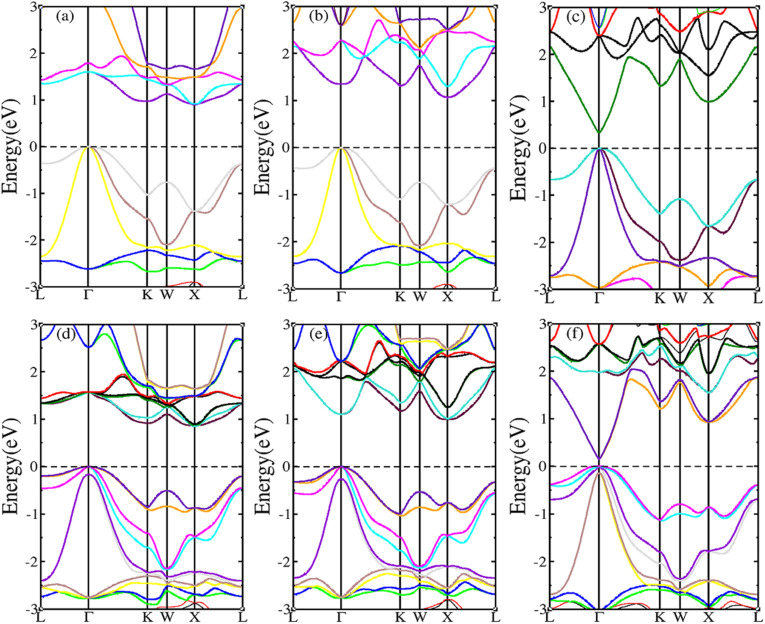
RhBiX (X = Ti, Zr, Hf) band structures with and without SOC. (a) RhBiTi (b) RhBiZr (c) RhBiHf. (d), (e) and (f) correspond with SOC respectively.

**Fig. 4:**
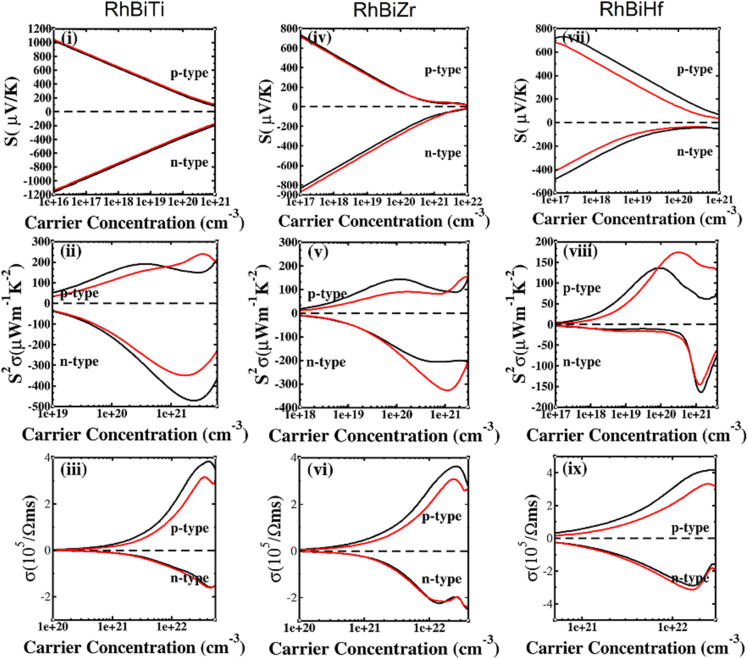
Seebeck (i, iv and vii), power factor (ii, v and viii), electrical conductivity (iii, vi and ix) calculated with mBJ (black line) and mBJ + SOC (red line) at room temperature.

The SOC effect can affect structure and properties of materials, hence, the electronic structures and thermoelectric properties of RhBiX (X = Ti, Zr, Hf) are investigated using mBJ + SOC. First, the band structures with mBJ and mBJ + SOC were compared, as shown in Fig. 3. Upon comparing band structures with and without SOC, it was identified that SOC reduces the band gaps, owing to the CBM moving toward lower energy. We find the sixfold degenerated VBM bands split into higher quadruple and lower twofold, and the corresponding values of spin–orbit splitting are shown in Table 2. These data indicate that the SOC effect has more infiuence on valence than conduction bands. Because the SOC effect modified the band structures, and the thermoelectric properties of RhBiX (X = Ti, Zr, Hf) are largely dependent on band structure. Therefore, mBJ and mBJ + SOC were utilized to examine thermoelectric properties, such as Seebeck coefficient, power factor, and electrical conductivity as a function of carrier concentration, as shown in Fig. 4. Because the variation trend of various properties is similar at different temperatures, only the room temperature (300 K) is presented in Fig. 4. The results demonstrate that SOC has an adverse effect on Seebeck coefficient, power factor, and power factor, however, the influence of P-type is greater than that of n-type doping. This can be explained by the fact that SOC effect on valence bands is greater than that of conduction bands. Similar SOC effects on thermoelectric properties have been identified in other HH materials.^27,58,59^ Therefore, the SOC effect is considered in subsequent thermoelectric properties calculations conducted herein.

**Table 2:** The relaxed equilibrium lattice constants *a* (Å), band gaps without SOC *E*_1_ (eV) and with SOC *E*_2_ (eV), the spin–orbit splitting energy Δ (eV)

	*a*	*E*_1_	*E*_2_	Δ
RhBiTi	6.265	0.906	0.841	0.166
RhBiZr	6.444	1.071	0.981	0.263
RhBiHf	6.406	0.333	0.157	0.144

## 5. Thermoelectric properties

**Fig. 5:**
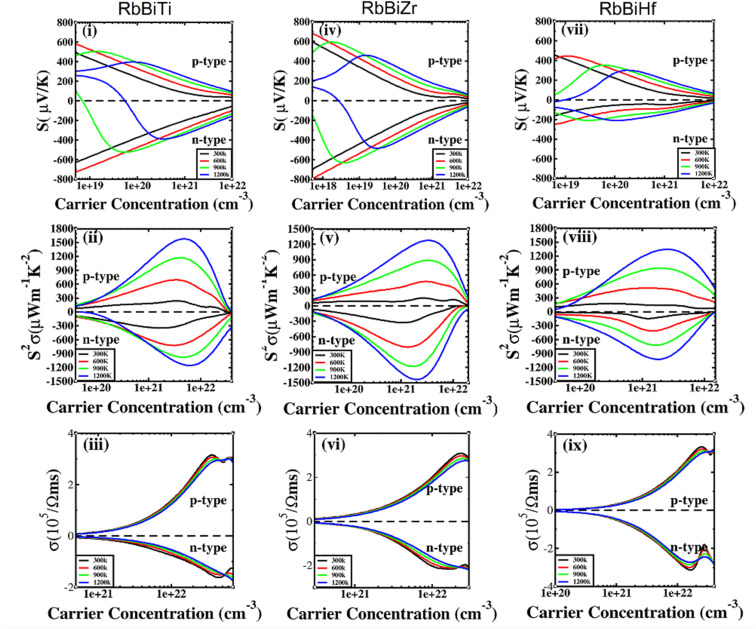
Thermoelectric properties of RhBiX (X = Ti, Zr, Hf) as a function of carrier concentration at different temperatures: (i, iv, vii) Seebeck coefficient, (ii, v, viii) power factor, and (iii, vi, ix) electrical conductivity.

The electronic transport properties of RhBiX (X = Ti, Zr, Hf) were investigated by the BoltzTraP code. The calculated thermoelectric properties such as Seebeck coefficient, power factor and electrical conductivity are discussed in Fig. 5. Although relaxation time constant (*τ*) depends on the doping level and temperature of RhBiX (X = Ti, Zr, Hf), to determine the thermoelectric parameters of the material, we use a constant relaxation time throughout the calculation. Referring to the XTaZ (X = Pd, Pt and Z = Al, Ga, In)^60^ and NiTiSn,^61^ we set the relaxation time *τ* = 1 × 10 ^−15^ s.

In Fig. 5(i), (iv) and (vii), the Seebeck coefficient is presented as a function of carrier concentration at different temperatures. Seebeck coefficient decreased with increasing carrier concentration at different temperatures. When the carrier concentration was fixed, the Seebeck coefficient increased with temperature. If the bipolar effect at high temperatures was neglected, for RhBiTi, n-type doping at each given temperature was higher than that of p-type doping. At 900 K, the highest Seebeck coefficients were 499 and −521 μV K^−1^ at p- and n-type doping concentrations of 1.4 × 10^19^ and 5.1 × 10^19^ cm^−3^, respectively. Furthermore, at 1200 K, the highest Seebeck coefficients were 389 and −390 μV K^−1^ at p- and n-type doping concentrations of 8.4 × 10^19^ and 3.5 × 10^20^ cm^−3^, respectively. For RhBiZr, it belongs to n-type doping as RhBiTi. By observing the curve of Seebeck coefficients, we observed that the difference between n- and p-type doping was not significant. The highest Seebeck coefficients were 595 and −635 μV K^−1^ at p- and n-type doping concentrations of 1.7 × 10^18^ and 3.7 × 10^18^ cm^−3^, respectively, at 900 K, while the values were 454 and −489 μV K^−1^ at p- and n-type doping concentrations of 1.6 × 10^19^ and 3.3 × 10^19^ cm^−3^, respectively, at 1200 K. For RhBiHf, the Seebeck coefficient belonged to p-type doping, which was different from RhBiTi and RhBiZr. The highest values of Seebeck coefficients were 353 and −217 μV K^−1^ at p- and n-type doping concentrations of 6.2 × 10^19^ and 2.9 × 10^19^ cm^−3^, respectively, at 900 K, while the highest values were 296 and −214 μV K^−1^ at p- and n-type doping concentrations of 1.8 × 10^20^ and 1.1 × 10^20^ cm^−3^, respectively, at 1200 K. The range of the carrier concentration for which the maximum value of the Seebeck coefficient appeared was 1.7 × 10^18^–1.1 × 10^20^ cm^−3^.

In narrow-bandgap semiconductors, a large slope of the state density close to energy gap corresponds to a large effective mass, which could induce a large Seebeck coefficient.^59,62^ The relationship between Seebeck coefficient and effective mass is 
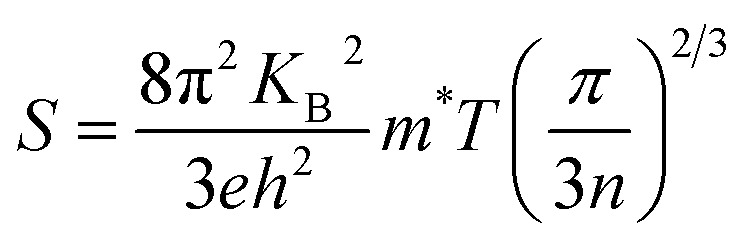
, where *K*_B_, *ℏ*, *e*, *T*, *n*, m* are the Boltzmann constant, Planck constant, electronic charge, absolute temperature, carrier concentration, and effective mass, respectively. The Seebeck coefficient was determined by the effective mass and carrier concentration at a constant temperature. Referring to calculated density diagrams shown in Fig. 1(b)–(d), from RhBiTi to RhBiZr and from RhBiZr to RhBiHf, the slope near the Fermi level decreased gradually, indicating that the corresponding Seebeck efficiency decreased gradually. The results of our calculations verify this prediction and agree with this, as shown in Fig. 5(i), (iv) and (vii). Generally, for RhBiX (X = Ti, Zr, Hf), the Seebeck coefficient of RhBiTi was better than the other two materials, and n-type doping was better than p-type doping.

The power factor is a useful parameter for balancing the thermal conductivity term. Theoretically, the optimal doping concentration, where the power factor exhibits the maximum value, can reduce the doping range in the experiment.^63^Fig. 5(ii), (v) and (viii) shows that power factor depends on the carrier concentration of RhBiX (X = Ti, Zr, Hf) at different temperatures. At a defined temperature, the power factor has a maximum peak with an increase in carrier concentration. At a fixed concentration, the power factor increased with temperature. We calculated the power factor values corresponding to the optimal doping level, which are shown in Table 3. Moreover, the power factor presents the highest values for p- and n-type doping in a range (0.3–6.3) × 10^21^ cm^−3^ at different temperatures. RhBiTi presents n-type doping at <600 K, while p-type doping at >600 K. RhBiZr presents n-type doping at different temperatures. RhBiHf shows an p-type doping with power factor values lower than the values of RhBiTi and RhBiZr at the same temperature. The higher value of power factor shows that RhBiTi is greater than RhBiZr and RhBiZr is greater than RhBiHf. The phenomenon is primarily related to state density near the Fermi level of RhBiX (X = Ti, Zr, Hf), and it is associated with a slow decline in DOS near the band edge^59,62^ from RhBiTi to RhBiZr and from RhBiZr to RhBiZr. A large slop of the state density near the Fermi level is crucial, implying heavy effective mass, which enhances the power factor.

**Table 3:** Maximum values of the power factor (μWm^−1^ K^−2^) of RhBiX (X = Ti, Zr, Hf) HH compounds at different temperatures and related optimal doping levels (× 10^21^ cm^−3^)

	Temperature	Doping level		Power factor	
		p-type	n-type	p-type	n-type
RhBiTi	300 K	3.5	1.7	237	−353
	600 K	3.4	3.3	696	−725
	900 K	4.2	4.5	1172	−990
	1200 K	4.9	6.3	1577	−1176
RhBiZr	300 K	2.6	1.1	145	−337
	600 K	3.0	1.4	477	−807
	900 K	3.4	1.7	887	−1181
	1200 K	3.3	2.1	1273	−1447
RhBiHf	300 K	0.3	1.2	177	−154
	600 K	1.2	1.5	502	−413
	900 K	2.0	1.7	933	−731
	1200 K	2.5	1.8	1352	−1030

In Fig. 5(iii), (vi) and (ix), the electrical conductivity as a function of the carrier concentration is shown at different temperatures for RhBiX (X = Ti, Zr, Hf) compounds. The electrical conductivity of RhBiHf (3.33 × 10^5^ Ωms^−1^) is greater than that of RhBiZr (3.09 × 10^5^ Ωms^−1^) and RhBiTi (3.06 × 10^5^ Ωms^−1^) at room temperature. The electrical conductivity decreased with an increase in temperature due to thermal collision.

**Fig. 6:**
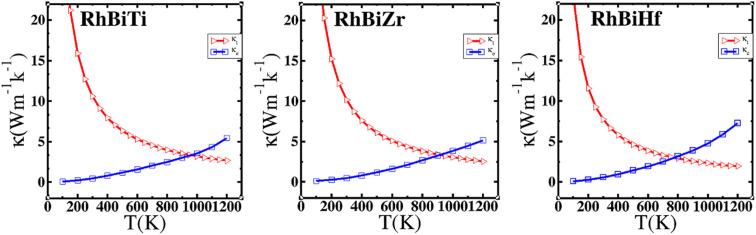
Variation of lattice thermal conductivity and electronic thermal conductivity of RhBiX (X = Ti, Zr, Hf) with temperature.

Thermal conductivity, which affects the efficiency of thermoelectric materials, primarily includes the *κ*_e_ and *κ*_ι_, which are related as *κ* = *κ*_e_ + *κ*_ι_. *κ*_e_ depends on carrier concentration at different temperatures, and it can be determined using the BoltzTraP code. Additionally, *κ*_ι_can be calculated using Slack’ s equation,^64^
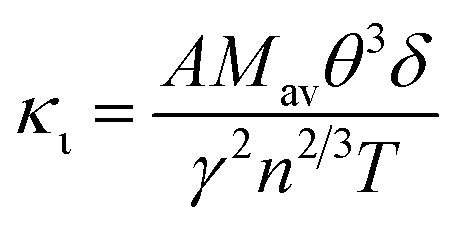
 where *M*_av_, *δ*, *n*, *T*, and *γ* represent the average atomic mass in the crystal, cubic root of average atomic volume, total number of atoms in the unit cell, absolute temperature, and Grüneisen parameter, respectively. Parameter *A*, which depends on factor *γ*, can be calculated as,^55^
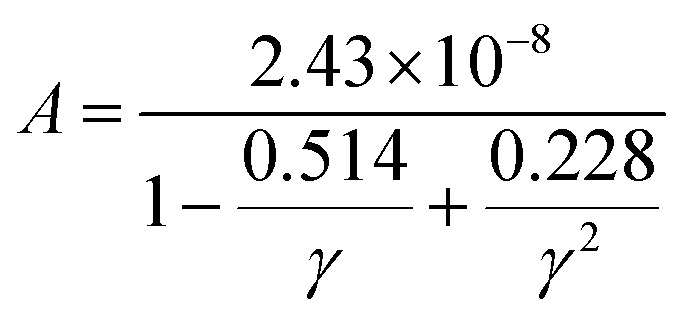
 where *γ* is the Grüneisen parameter, which can be calculated by Poisson's ratio, 
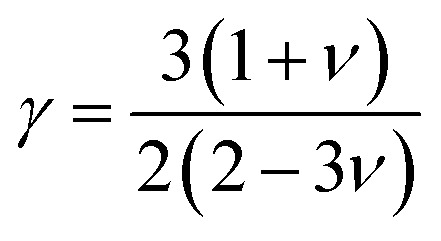
. Using the Slack equation defined, the temperature dependence of lattice thermal conductivity is calculated and plotted, as shown in Fig. 6. Furthermore, the lattice thermal conductivity of RhBiX (X = Ti, Zr, Hf) decreased with the increase of temperature. Small lattice thermal conductivity is one of the most favorable conditions for RhBiX(X = Ti, Zr, Hf) to produce high thermoelectric efficiency. Lattice thermal conductivities of RhBTi, RhBiZr, and RhBiHf are 10.60, 10.15, and 7.71 W mK^−1^ at 300 K, respectively. The result is consistent with a previous theoretical study, which shows the conductivities of 11.41, 12.45, and 10.46 W mK^−1^ for RhBTi, RhBiZr, and RhBiHf, respectively, at the same temperature.^65^ According to the Wiedemann–Franz equation (*κ*_e_ = *LσT*, *L* is the Lorentz factor), the electron thermal conductance graph was calculated as a function of temperature as shown in Fig. 6. The decreasing trend of thermal conductivity with increasing temperature was evident because phonon scattering increased with temperature. Based on the calculated results, lattice thermal conductivity decreased with the temperature increase, while electronic thermal conductivity increased as temperature increased. When the temperature was >300 K, the lattice thermal conductivity changed slowly, and the curve appeared smooth, which indicated that RhBiX (X = Ti, Zr, Hf) compounds showed a good thermoelectric response. When the temperature reaches a certain value, lattice thermal conductivity identical electronic thermal conductivity. Specific temperature values of RhBiTi, RhBiZr and RhBiHf were 960, 900 and 780 K, respectively. At higher temperatures, thermal conductivity is predominantly contributed electronically. A similar phenomenon has been observed in TiNiSn.^66^

**Fig. 7:**
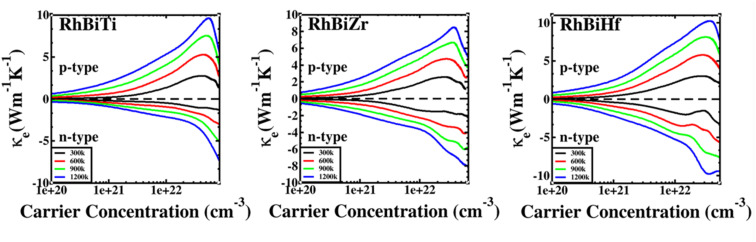
Variation of electronic thermal conductivity of RhBiX (X = Ti, Zr, Hf) with temperature.

The electronic thermal conductivity of RhBiX (X = Ti, Zr, Hf) depends on carrier concentration at different temperatures, as shown in Fig. 7. Additionally, the electronic thermal conductivities of the three materials show the same variation with the carrier concentration. When the carrier concentration was 5.5 × 10^22^ cm^−3^, the maximum electron thermal conductivity was observed. The electronic thermal conductivity increased with the temperature from 300 to 1200 K. A similar phenomenon has been observed in other HH alloys,^35,55^ thereby confirming the accuracy of our calculations.

**Fig. 8:**
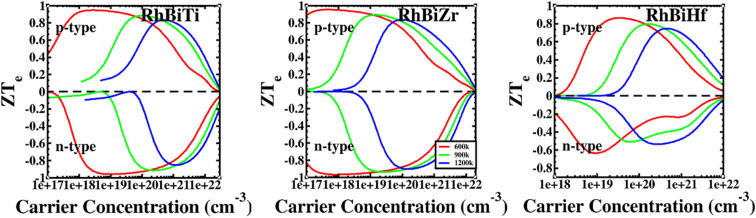
The ratio of RhBiX (X = Ti, Zr, Hf) as a function of carrier concentration at different temperatures.

The conversion efficiency of thermoelectric materials in applications is determined by the dimensionless figure of merit *ZT*, which is defined as^67^*ZT* = *ZT*_e_ × *κ*_e_/(*κ*_e_ + *κ*_ι_). The thermal conductivity of electrons increased at high temperatures due to thermally excited electron–hole pairs. Furthermore, a decrease in the mean free path (Lph) of phonons led to a sharp decrease in lattice thermal conductivity. Fig. 6 shows that at higher temperatures, thermal conductivity is predominantly contributed electronically. Therefore, it is assumed that when the temperature is over 600 K, the lattice thermal conductivity value is ignored. The ratio *ZT*_e_ = *S*^2^*σT*/*κ*_e_ is independent of the relaxation time *τ* because the electronic thermal conductivity increased with temperatures. A plot of *ZT*_e_ is presented as a function of carrier concentration for temperatures greater than 600 K, as shown in Fig. 8.

If we could not consider the bipolar effect, carrier concentration was fixed. Afterward, *ZT*_e_ increased with *T*. Furthermore, if the temperature was fixed with the increase of carrier concentration, first, *ZT*_e_ increased, reached a maximum value, and subsequently decreased gradually. The maximum value of *ZT*_e_ occurs at the carrier concentration at approximately 10 × 10^20^ cm^−3^ at >600 K. The highest values were 0.91, 0.94, and 0.79 for RhBiTi, RhBiZr, and RhBiHf when temperature at 900 K, respectively, showing higher values of *ZT*_e_ compared to ZrRhSb, which had been reported.^68^ For RhBiTi and RhBiZr, the n-type doping was slightly larger than the p-type doping, while for RhBiHf, p-type doping was greater than n-type doping. The estimates of RhBiTi *ZT* values are extremely similar to those previously studied for RhTiBi at high temperatures.^69^ For RhBiX (X = Ti, Zr, Hf) compounds, because the influence of lattice thermal conductivity was neglected, the thermoelectric superiority value obtained might be slightly overestimated. However RhBiX (X = Ti, Zr, Hf) are comparable to several other Heusler alloys, such as TiNiSn,^70^ and NiTZ.^71^ Thus, RhBiX (X = Ti, Zr, Hf) are favorable candidates for utilization as p- or n-type elements in TE devices. Moreover, RhBiX (X = Ti, Zr, Hf) compounds are new materials with multiple unknown properties that must be investigated in future research. Additionally, the results of the calculations and trends can be used as a reference for theory and experiments.

**Fig. 9:**
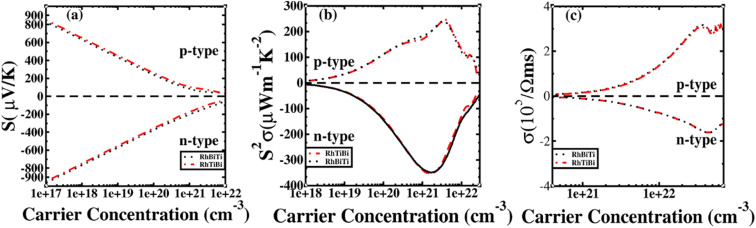
The Seebeck coefficient (a), power factor (b), and electrical conductivity (c) as a function of carrier concentration at room temperatures. Black dashed line represents RhBiTi,. red dashed line represents RhTiBi.

The potential was confirmed to predict the electronic properties for HH materials with 8 or 18 valence electrons per primitive cell, in which their electronic structure is related to the atom occupying the 4c position.^72^ Therefore, RhBiTi was used as an example to investigate the differences in thermoelectric properties between RhBiTi and RhTiBi, in which the position of 4C are all Rh. The Seebeck coefficients, power factors and electrical conductivity varying with carrier concentration at room temperature (300 K) are shown in Fig. 9. It can be observed that almost no difference exists in the various thermoelectric properties of both structures, thus verifying the conclusions in ref. 72. Because the same atoms occupying the 4c position, the electronic structures and thermoelectric properties of RhBiTi and RhTiBi are identical.

## 6. Conclusion

Based on the first-principles and Boltzmann transport theory, the electronic structure, mechanical and thermoelectric properties of RhBiX (X = Ti, Zr, Hf) were investigated. The lattice parameters which optimized were in good agreement with related research. The electronic structures showed that RhBiTi and RhBiZr are indirect bandgap semiconductors, while RhBiHf is a direct bandgap semiconductor. In the phonon spectrums of RhBiX (X = Ti, Zr, Hf), the absence of imaginary or negative frequency confirms the dynamical stability. The narrow band characteristics indicate that RhBiX (X = Ti, Zr, Hf) are expected to be good thermoelectric materials. The variations of the thermoelectric parameters of RhBiX (X = Ti, Zr, Hf), such as Seebeck coefficient, power factor, electron thermal conductivity, and thermoelectric optimum value are studied for the first time with carrier concentration, temperature, and doping. The lattice thermal conductivity at room temperature is in good agreement with the theoretical study. In addition, the calculated thermoelectric optimum values were 0.91, 0.94, and 0.79 for RhBiTi, RhBiZr, and RhBiHf at 900 K, respectively, which show that RhBiX (X = Ti, Zr, Hf) alloys are promising thermoelectric materials for a wide range of temperature.

## Conflicts of interest

There are no conflicts to declare.

## Supplementary Material
